# Embarrure suite à l'utilisation de la têtière de Mayfield chez l'adulte: à propos d'un cas et revue de la literature

**DOI:** 10.11604/pamj.2016.24.129.8367

**Published:** 2016-06-09

**Authors:** Mohamed Moutaoukil, Mustapha Bensghir, Soukaina Eddik, Abdelhamid Jaafari, Redouane Ahtil, Mohammed Meziane, Charki Haimeur

**Affiliations:** 1 Service d'Anesthésiologie, Hôpital Militaire Med V, Université Souissi Med V, Rabat, Maroc

**Keywords:** Embarrure, têtière de Mayfield, neurochirurgie, hypertension intracrânienne, Depressed skull fracture, mayfield headrest, neurological surgery, intracranial hypertension

## Abstract

Un grand nombre d'interventions neurochirurgicales nécessitent l'utilisation d'une têtière à broches pour immobiliser la tête du patient. Nous rapportons le cas d'une embarrure chez un adulte secondaire à l'utilisation de la têtière de Mayfeild. Le diagnostic a été posé en postopératoire d'une résection chirurgicale d'un médulloblastome par une tomodensitométrie cérébrale. Plusieurs facteurs semblent contribuer à augmenter le risque de complications dues à l'utilisation de la têtière de Mayfield. Les mesures de prévention sont discutées à travers une revue de littérature.

## Abstract (en)

Many neurosurgical procedures involve the use of a pin-type headrest to immobilize the patient's head. We report the case of depressed skull fracture in an adult patient secondary to the use of Mayfield headrest. The diagnosis was based on postoperative CT scan of the brain following surgical resection of medulloblastoma. Several factors seem to increase the risk of complications due to Mayfield headrest use. Preventive measures are outlined in our literature review.

## Introduction

Beaucoup de procédures en neurochirurgie exigent une immobilisation stricte de la tête tout au long de l'acte opératoire. La têtière à broches type Mayfield est la plus utilisée dans ce contexte. Elle procure une fixation rigide du crâne et du rachis cervical dans des positions particulières sans restreindre le champ opératoire [1]. La rareté des rapports sur les complications liées à l'utilisation de ce dispositif de fixation a généré un faux sentiment de sécurité liée à son utilisation. Nous relatons par cette observation la survenue d'une embarrure chez un adulte secondaire à l'utilisation de la têtière de Mayfield.

## Patient et observation

**Figure 1:**
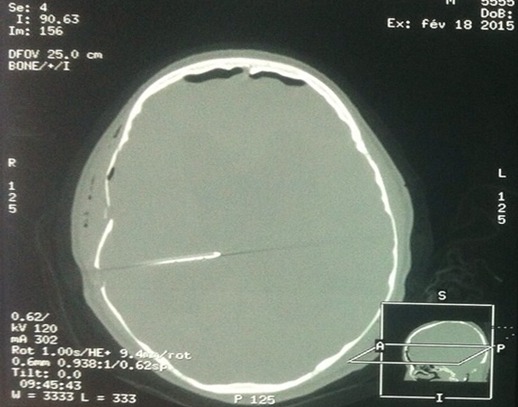
Fenêtre osseuse objectivant une fracture à cheval entre la suture sagittale et le trou de trépan de la DVP avec enfoncement de la table externe sur la table interne de la voute crânienne associée à une discrète pneumocéphalie en regard

**Figure 2:**
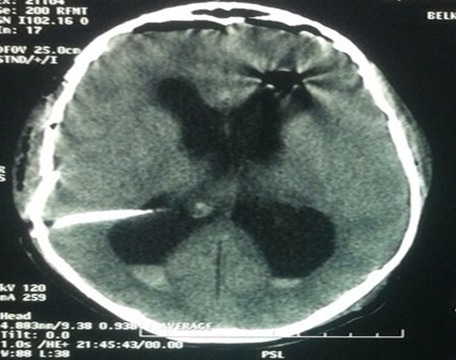
Fenêtre parenchymateuse objectivant la fracture déplacée de l'os pariétal

Un patient âgé de 17 ans, sans antécédents et notamment pas de notion de traumatisme crânien antérieur était admis à l'hôpital pour prise en charge d'une tumeur du quatrième ventricule. L'examen clinique trouvait un patient conscient sans déficit neurologique, présentant depuis un an des céphalées à prédominance matinale sans vomissements associées ni baisse de l'acuité visuelle. Le tableau clinique s’était aggravé cinq mois après par l'apparition des troubles de la marche à type de pas irréguliers, marche ébrieuse avec polygone de sustentation élargi. La TDM et l'IRM cérébrale objectivaient une tumeur du quatrième ventricule se rehaussant de façon homogène après injection de produit de contraste avec une hydrocéphalie triventriculaire très importante. Le patient a subi initialement une dérivation ventriculo-péritonéale puis une semaine après le patient était reprogrammé pour un geste radical. Après une antibioprophylaxie par 2g de céfazoline, l'anesthésie était induite par 400 mg de thiopental, 300 mg de fentanyl et 50 mg de rocuronium puis entretenue en ventilation contrôlée par une anesthésie totale intraveineuse à objectif de concentration par propofol et rémifentanil. La tête a été fixée par le cadre de Mayfield avec une force de serrage de 60 pounds puis le patient a été mis sur le décubitus ventral. Aucun incident per opératoire n'a été noté avec exérèse subtotale de la tumeur. L'examen extemporané de la pièce opératoire était en faveur d'un médulloblastome. A la fin de l'intervention, en retirant les broches du cadre, l'orifice de la peau correspondant à la broche pariétale droite avait commencé à saigner avec à la palpation une dépression de l'os pariétal en regard faisait immédiatement suspecter une fracture de l'os pariétal. La petite plaie du cuir chevelu a été suturée par des points de suture. Une tomodensitométrie cérébrale a objectivé une fracture déplacée de l'os pariétal avec discrète pneumocéphalie en regard et sans lésions intracrâniennes associées (Figure 1, Figure 2). Le patient était extubé au bout de 30 min puis transféré dans le service de neurochirurgie après 48 heures en raison d'une évolution favorable. Les suites étaient favorables et le patient rejoignait son domicile au huitième jour.

## Discussion

Depuis sa conception par Gardner, plusieurs modifications ont été apportées aux têtières à trois broches [1]. Actuellement, la têtière de Mayfield est la plus utilisée dans le monde entier. Elle est conçue pour stabiliser fermement la tête durant la craniotomie avec minimum de lésions du scalp permettant ainsi la réalisation des actes opératoires de longue durée sans restreindre le champ opératoire [2]. Malgré son utilisation large en neurochirurgie, on trouve qu'un nombre limité de rapports dans la littérature concernant les complications liées à son utilisation aussi bien chez l'adulte que chez l'enfant (Tableau 1). Parmi ces complications, on trouve: l'infection des sites d'insertion des broches, pseudo-anévrysme de l'artère temporale superficielle, fistule artério-veineuse méningée moyenne, embolie gazeuse veineuse qui est rarement relatée, et surtout chez des patients opérés en position assise, ou lorsque la têtière a été enlevée avec la tête au-dessus du niveau du cœur et les fractures du crâne isolées ou associées à une pneumocéphalie ou à une hémorragie intracérébrale. Cette dernière complication a été décrite essentiellement chez les enfants et elle est liée probablement à la faible épaisseur du crâne [1, 3–12]. Plusieurs facteurs ont été relevés comme étant responsables de ses complications chez l'adulte. Ils comprennent le jeune âge, une application défectueuse des broches, une pression excessive appliquée pendant l'emplacement des broches, les opérations de longue durée, la prise au long cours des antiépileptiques et les pathologies fragilisant l'os comme l'insuffisance rénale chronique avec une hyperparathyroïdie secondaire.

**Tableau 1:** Cas de complications de la têtière à broches publiés

Auteurs	Age/sexe	Chirurgie	Type de la têtière	Complication	Traitement	Facteurs de risque
Matouk CC [2]	79/ M	décompression cervicale postérieure pour MCA	Mayfield	Fracture du crâne + HED	Conservateur	IRC+ antiépileptique
Yan HJ [6]	15/ M	craniotomie suboccipitale pour papillome du plexus choroïde	Mayfield	Fracture du crâne + HED	Evacuation de l'HED	Localisation de la broche sur la suture coronale
Erbayraktar [10]	23/M	Craniotomie frontale pour récidive d'un adénome pituitaire	Multi-Poise	Fracture du crâne + HED	Evacuation de l'HED	Application défectueuse de la têtière
Sade [11]	24/M	Craniotomie frontopariétale pour méningiome	Mayfield	Fracture du crâne + HED	Evacuation de l'HED	Elévation chronique de la PIC
Lee [12]	38/ F	foraminotomies cervicale Postérieure	Mayfield	Fracture du crâne + HED	Evacuation de l'HED	Asymétrie de l’épaisseur du crane
Bindra [16]	40/ F	craniotomie suboccipitale rétromastoide pour schwannome de l'acoustique	Mayfield	Bilatéral HED	Evacuation de l'HED	Aucun
Moumoulidis [17]		craniotomie frontale pour glioblastome multiforme	Mayfield	Fracture de la table externe et interne du sinus frontal	Conservateur	Localisation de la broche sur le sinus frontal
Tang CT [18]	15/M	craniotomie suboccipitale	Mayfield	Fracture du crâne + HED	Evacuation de l'HED	Elévation chronique de la PIC
Jha NK [19]	22/M	craniotomie pour tumeur du quatrième ventricule	Mayfield	Fracture temporale bilatérale avec HED gauche	Evacuation de l'HED	Elévation chronique de la PIC
Notre cas	17/M	craniotomie suboccipitale pour tumeur du quatrième ventricule	Mayfield	Fracture du crane	conservateur	Elévation chronique de la PIC

**Age:** exprimé en années; **M:** Homme; **F:** Femme; **HED:** Hématome extradural; **PIC:** pression intracranienne; **IRC:** Insuffisance rénale chronique; **MCA:** Myélopathie cervicoarthrosique

Chez notre patient, de multiples facteurs ont abouti à cet incident: premièrement; l’évolution à longue terme d'une hypertension intracrânienne a probablement aboutit à un amincissement de la voute crânienne; comme cela a été démontré par Sade and Mohr chez un patient âgé de 24 ans qui présentait une histoire chronique d'hypertension intracranienne et qui a subi une craniotomie fronto-pariétale pour résection d'un méningiome para sagittal. Six heures après l'intervention, le patient a présenté un hématome extradural sous-jacent à une fracture du crâne au site d'insertion de la broche de la têtière de Mayfield [11]. Secondairement, la localisation de point d'insertion de pin de la têtière à proximité de la suture sagittale en haut et le trou de trépan de la dérivation ventriculo-péritonéale en bas a contribué à fragiliser l'os pariétal d'avantage ce qui a facilité la survenue de cette complication. Pour diminuer l'incidence de ces complications, il a été proposé: d’éviter, d'insérer les broches de la têtière dans les zones fragiles du crâne, y compris l’écaille temporale, le sinus frontal et scissure coronale [13, 14]; d’éviter, d'utiliser les têtières à broches chez les enfants âgés moins de 5 ans et chez les adultes présentant l'un des facteurs décrits ci-dessus sauf dans les interventions nécessitant une immobilisation stricte de la tête (stéréotaxie, neuronavigation…) [15]; d’évaluer l’épaisseur du crâne sur le scanner et par conséquent adapter la taille des broches et la force de serrage [7]. Chez l'adulte La force de serrage recommandée pour la têtière de Mayfield est de l'ordre de 60 à 80 pounds, tandis qu'il n'existe pas de recommandations précises pour le niveau de force à utiliser chez les enfants. Certains proposent une force de 5 pounds pour les enfants âgés entre 6 et 12 mois; 10 pounds pour les enfants âgés entre 12 mois et 2 ans; 20 pounds pour les enfants âgés entre 2 et 5 ans et pour les enfants âgés entre 5 et 12 ans une force de 30 pounds peut être utilisée [6]; Le retrait des broches de la têtière doit être fait en position de décubitus [4].

## Conclusion

Les complications liées à l'utilisation de la têtière à broches sont souvent sous-estimées. Elles peuvent être dévastatrices. Elles doivent être connues par tous les cliniciens afin de prendre les mesures de précaution nécessaires chez les patients à risque et permettre en cas de survenue une prise en charge précoce et adaptée.
